# The Interplay Between Immune Response and Bacterial Infection in COPD: Focus Upon Non-typeable *Haemophilus influenzae*

**DOI:** 10.3389/fimmu.2018.02530

**Published:** 2018-11-05

**Authors:** Yu-Ching Su, Farshid Jalalvand, John Thegerström, Kristian Riesbeck

**Affiliations:** ^1^Clinical Microbiology, Department of Translational Medicine, Faculty of Medicine, Lund University, Malmö, Sweden; ^2^Department of Biology, Centre for Bacterial Stress Response and Persistence, University of Copenhagen, Copenhagen, Denmark

**Keywords:** airway, COPD, exacerbation, immune response, infection, inflammation, non-typeable *Haemophilus influenza*

## Abstract

Chronic obstructive pulmonary disease (COPD) is a debilitating respiratory disease and one of the leading causes of morbidity and mortality worldwide. It is characterized by persistent respiratory symptoms and airflow limitation due to abnormalities in the lower airway following consistent exposure to noxious particles or gases. Acute exacerbations of COPD (AECOPD) are characterized by increased cough, purulent sputum production, and dyspnea. The AECOPD is mostly associated with infection caused by common cold viruses or bacteria, or co-infections. Chronic and persistent infection by non-typeable *Haemophilus influenzae* (NTHi), a Gram-negative coccobacillus, contributes to almost half of the infective exacerbations caused by bacteria. This is supported by reports that NTHi is commonly isolated in the sputum from COPD patients during exacerbations. Persistent colonization of NTHi in the lower airway requires a plethora of phenotypic adaptation and virulent mechanisms that are developed over time to cope with changing environmental pressures in the airway such as host immuno-inflammatory response. Chronic inhalation of noxious irritants in COPD causes a changed balance in the lung microbiome, abnormal inflammatory response, and an impaired airway immune system. These conditions significantly provide an opportunistic platform for NTHi colonization and infection resulting in a “vicious circle.” Episodes of large inflammation as the consequences of multiple interactions between airway immune cells and NTHi, accumulatively contribute to COPD exacerbations and may result in worsening of the clinical status. In this review, we discuss in detail the interplay and crosstalk between airway immune residents and NTHi, and their effect in AECOPD for better understanding of NTHi pathogenesis in COPD patients.

## Introduction

The lungs are vital organs involved in gas exchange between the vascular system and the external environment, thus they are greatly exposed to the environment-derived microorganisms, including fungi, viruses, and bacteria. The bronchial tree and parenchymal tissues of the lungs, that until recently were considered as sterile, are colonized by phylogenetically-diverse microbes. The genera of *Firmicutes, Bacteroidetes*, and *Proteobacteria* are the most common phyla identified and represent 60% of the total bacterial microbiome in the healthy airway (1, 2). The majority of the lung microbiota belongs to the normal flora that play an important role in the pulmonary epithelial integrity, colonization resistance, and homeostasis of the immune system in the respiratory tract (3). A small fraction of them are, however, potentially pathogenic microorganisms that are involved in a variety of lung diseases, as exemplified by the genus *Haemophilus*. Non-typeable *Haemophilus influenzae* (NTHi) is a Gram-negative coccobacillus that are commonly residing in the human airways. Uniquely and yet unexplained, NTHi is a commensal when colonizing the nasopharynx or throat, but pathogenic in the lower airways triggering a robust inflammatory response [for reviews see (4, 5)]. NTHi is considered a potential opportunistic pathogen as it frequently infects the lower respiratory tract of lungs with structural damage as a consequence of non-infectious lung diseases or mechanical injuries. Moreover, NTHi occasionally causes bronchitis and pneumonia (6). In addition, lower airway colonization by NTHi has been associated with disease progression of several more or less non-infectious lung diseases such as bronchiectasis (7), cystic fibrosis (8), interstitial lung diseases (9, 10), but mostly in chronic obstructive pulmonary disease (COPD) (11, 12). COPD is a severe inflammatory lung disease characterized by airflow limitation with a range of pathological changes. Both genetics and environmental factors trigger the onset of COPD, however, microbes including NTHi play an important role in the acute exacerbations. This review describes the disease progression of COPD in the context of host immune-interactions linked to NTHi, and the overall impact in disease exacerbation.

## The pathophysiology of COPD

COPD is the third leading cause of morbidity and mortality worldwide expected to affect more than 210 million people by 2030 (13, 14). According to the Global Initiative for Chronic Obstructive Lung Disease (GOLD), COPD is a pulmonary disease that is manageable, but significant exacerbations and co-morbidities may, however, contribute to the overall severity in individual patients (15). COPD is characterized by chronic airflow limitation of the peripheral airways with a range of pathological changes in the lung that are not fully reversible, and usually become progressively worse over time. The progression of COPD is associated with an abnormal inflammatory response of the lung to noxious particles or gases.

From a pathological point of view, COPD comprises a group of pulmonary abnormalities related to the inflammatory reaction of the airways, alveoli, and pulmonary vessels (16–19). These include (i) pulmonary emphysema, (ii) chronic bronchitis, and (iii) disease in the small airways. The pulmonary abnormalities progressively affect all parts of the lung, resulting in increased resistance of the conducting airways and thus chronic airflow obstruction that eventually will lead to a declined lung function. **Emphysema** is a permanent loss of elastic lung recoil caused by elastolytic destruction and enlargement of the alveolar wall distal to the terminal bronchioles. This consequently results in the loss of alveolar attachments to the small airways and thus limitation of airflow and gaseous exchanges. **Chronic bronchitis** is characterized by consecutive and chronic cough with expectorations that last for more than 3 months within 2 years. It is associated with inflammation of the bronchial walls with increased inflammatory infiltrates, hyperplasia of goblet cells, hypertrophy of tracheobronchial submucosa, increased mucous secretion and, finally, dilatation of the airway ducts (airways of about 2–4 mm in internal diameter). The majority of the ciliated epithelium lining the airways are also either compromised or dysfunctionnal, and may be replaced by non-ciliated squamous epithelial cells. **Small airway diseases**, on the other hand, involve hyperplasia and metaplasia of mucosal glands and goblet cells, hypersecretion of intraluminal mucus, macrophage bronchiolitis, and accumulation of lymphocytes in the small bronchioles (airways of ~2 mm or less in diameter and terminal bronchioles). In addition, distortion, fibrosis, stenosis, tortuosities, hyperplasia, and hypertrophia of the small airway smooth muscles also contribute to the loss of elasticity in the lung parenchyma. Although COPD mainly affects the lungs, it also produces significant extrapulmonary consequences as a results of an escalated inflammatory response orchestrated by airway cells and immune mediators (20, 21). The co-morbidities are commonly seen in COPD patients despite the actual mechanism responsible for the systemic inflammation remains to be elucidated.

## The risk factors of COPD and consequences for airway function

The development of COPD is multifactorial, with cigarette or tobacco smoking being the primary cause of COPD (22, 23). Other risk factors that may promote the onset and progression of COPD includes prolonged occupational exposure to particles/gases in mining and textile industries, air pollution resulting from biomass combustion, and bronchial hyperresponsiveness (16, 18, 24). The variability of COPD incidences among smokers is also explained by a genetic pre-disposition, such as α1-antitrypsin deficiency and cutis laxa [mutation of the elastin gene *(ELN)*] (25, 26). The α1-antitrypsin deficiency is caused by deleterious homozygous mutations in SERPINA1 which contributes to 1–2% of COPD cases. The deficiency results in increased neutrophil elastase activity that ultimately leads to the degradation and collapse of the alveoli. Importantly, meta-analyses of genome-wide association studies (GWAS) and other genotyping studies have revealed that multiple single nucleotide polymorphism (SNPs) in at least 34 genes from different pulmonary genomic loci are associated with COPD susceptibility (27–30).

Airway epithelium exposed to cigarette or tobacco smoke has compromised tight junctions and delayed epithelial wound repair (31–34). Moreover, cigarette smoke alters basal cell differentiation and subepithelial extracellular matrix (ECM) composition, and thus causes airway remodeling (i.e., goblet cell hyperplasia and small airway squamous metaplasia) (35–37). This results in mucus hypersecretion, impaired mucocilliary clearance, and airway obstruction. Tobacco or cigarette smoke also enhances proliferation and ECM deposition by activating the extracellular signal related kinase (ERK) and the p38 signaling pathway (38). The alteration of major ECM components are widespread in all lung compartments in COPD patients with a total increase of type I and III collagens, fibronectin, and laminin in parallel with reduced concentrations of proteoglycans, perlecan decorin, versican, biglycan, tenascin and elastin (39, 40). Cigarette induced-overexpression of matrix metalloproteases (MMPs-1, 2, 7, 9, 12, and 28) and elastase has also been reported, and may contribute to the airway tissue destruction and fibrosis (41–43). In addition, harmful volatile chemicals derived from cigarette smoke (i.e., acetaldehyde, acrolein, and crotonaldehyde) are prone to form carcinogen adducts with DNA and various proteins (i.e., apoliprotein E and surfactant protein A). They also dysregulate airway epithelial ion transport, disrupt the phagocytic activity of airway phagocytes, and diminish the airway surface liquid volume (44–46).

Numerous proteomics and transcriptomic analyses have unveiled the crucial impact of cigarette or tobacco smoke and COPD disease progression on airway gene expression (47, 48). The differential gene expression studies were done using COPD experimental models or clinical samples [i.e., bronchial epithelial cells, sputum, plasma, blood, and bronchoalveolar lavage (BAL) fluid]. Collectively, most of the altered genes are involved in oxidative stress, xenobiotic metabolism, antioxidant responses, DNA repair, ECM remodeling, inflammatory responses, and immune defenses, which the latter two are our major interest of discussion in this review. The omics data aid in the increased knowledge of molecular mechanisms in COPD. They may reflect the dynamic response and attempts by the airway epithelial cells to repair the cytotoxic injury primarily triggered by inhaled irritants. Deleterious and irreversible alterations occurring and interfering with the airway epithelial homeostasis and immune defense may promote COPD development and progression. Notably, gene alterations in phagosomal- and leukocyte transendothelial migration pathways (LTM) are significantly correlated with the level of T cells and airway obstruction in smokers (49). The LTM, however, were found to be further dysregulated in COPD patients. Hence, in addition to clinical/physiology variables, a number of gene products with significant differential gene expression may be targeted as specific proteomic signatures or biomarkers for early COPD detection, patient monitoring, disease subgrouping, and finally treatment selection (50, 51).

## Alteration of airway gene expression and immune response in COPD

### Effects of tobacco or cigarette smoking

**Figure 1:**
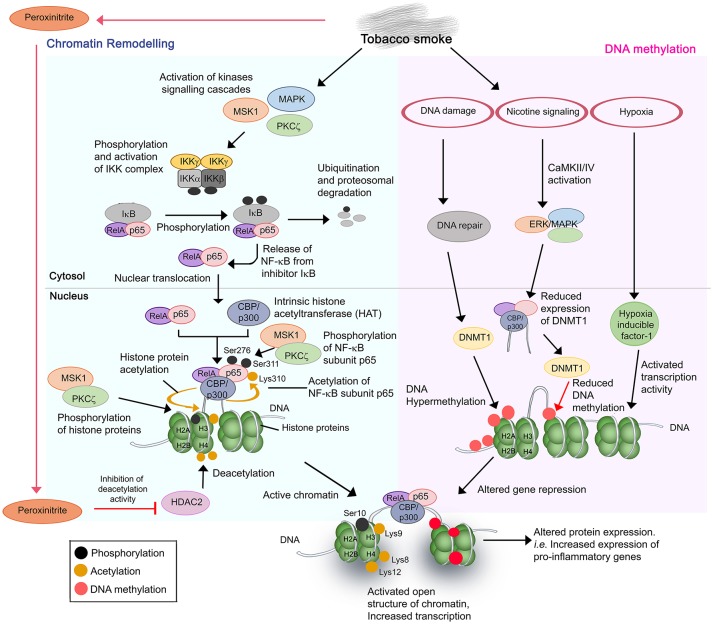
Cigarette and tobacco smoke has several effects on gene regulation. Nicotine and other compounds in the smoke alter gene expression by two pathways, firstly, chromatin remodeling **(Left)** and secondly, DNA methylation **(Right)**. Chromatin remodeling involves activation of kinases signaling pathways, activation and nuclear translocation of transcription factor NF-κB (RelA/p65), and complex formation with CBP/p300 on specific DNA sites. CBP/p300 is intrinsically a histone acetyltransferase (HAT). Subunit p65 is further phosphorylated at Ser276 and Ser311, respectively, by MSK1 and PKCζ, whereas CBP acetylates p65 at Lys310. The phosphorylation and acetylation enhance the interaction within the NF-κB/CBP/p300 complex while stabilizing the DNA binding of NF-κB. The complex of NF-κB/CBP/p300 then modifies the histones through CBP-mediated acetylations of histone H3 (at Lys9) and H4 at Lys 8 and Lys12, and phosphorylation of H3 at Ser10 by MSK1 and PKCζ. This results in the structure change of chromatin, from a condensed structure (repressed) to an activated open conformation. The transcription of target genes is therefore increased. In the second mechanism, several side effects resulting from cigarette smoking such as DNA damage and nicotine signaling could trigger the hypermethylation or decreased methylation of target DNA. This may lead to DNA methylation anomalies and thus altered DNA expression. Resulting hypoxia due to high concentrations of carbon monoxide also contributes to altered gene expression. The aberrant gene expression by cigarette smoke mostly occurs in pro-inflammatory genes with resulting increased production of inflammatory mediators, and amplified inflammation in the COPD lung upon exposure.

Tobacco or cigarette smoke regulates airway gene expression via two main mechanisms, by altering the status of (i) chromatin remodeling, and (ii) DNA methylation of the target genes (Figure 1) (52–54).

Chromatin remodeling is a result of a disrupted balance in histone acetylation/deacetylation (55). Excessive activation of more than 20 transcription factors including NF-κB, and lipoprotein peroxidation products (peroxinitrite, acrolein, and 4HNE from tobacco smoking) contributes to such anomaly. NF-κB is a key inflammatory and redox-sensitive transcription factor that plays a direct role in cigarette smoke-induced airway inflammation. NF-κB has been described as a “smoke-sensor” due to its sensitive activation by tobacco residues (56). Stimulation of multiple signaling cascades [p38 mitogen-activated protein (MAPK) kinases, mitogen and stress-activated kinase 1 (MSK1), protein kinase C zeta (PKCζ), and IκB kinase (IKK) complex (IKKα, IKKβ, and NEMO)] by tobacco residues promotes the activation and nuclear translocation of transcription factor NF-κB RelA/p65 (54, 57–64). This is followed by a complex formation of NF-κB/CBP-p300 [coactivator, CREB-binding protein (CBP) or CBP/p300] at target DNA sequences. It should be noted that CBP/p300 also has intrinsic histone acetyltransferase (HAT) activity. Subsequent acetylation and phosphorylation of the subunit p65 in the NF-κB/CBP-p300 complex by the activated MSK1/PKCζ-signaling pathways (and other 11 different kinases), and CBP/p300, respectively, are required for the full activation of NF-κB (57, 60, 63). This enhances the DNA binding affinity of the complex. Histones H3 and H4 in the chromatin complex of target sequences are then being acetylated (histone H3 at Lys9; H4 at Lys8 and Lys12) and phosphorylated (histone H3 at Ser10) by the subunit CBP of the NF-κB/CBP-p300 complex, and the activated MSK1 and PKCζ, respectively. The hyperacetylated core histones, however, fail to be neutralized or deacetylated by a dysfunctional histone deacetylase (HDAC2). Peroxinitrite nitrates the tyrosine residues of the HDAC2 and causes inhibition of activation and reduced expression of the protein. Of note, peroxinitrite is a by-product generated from the immune cell-derived nitrite oxide (NO) and reactive oxygen species (ROS) of cigarette smoke (65, 66).

Cigarette or tobacco smoke disturbs the DNA methylation status of target genes through several mechanisms. Firstly, DNA damage caused by cigarette smoke stimulates the DNA methyltransferase 1 (DNMT) to actively induce CpGs methylation at the damaged site (67). The hypermethylation is prone to introduce error of methylation in some target genes, resulting in reduced gene expression. Secondly, activation of nicotine signaling pathway by tobacco smoke causes CaMKII/IV and ERK/MAPK pathway activation that subsequently induces the activity of CBP to suppress the expression of DNMT1. This may result in reduced DNA methylation and thus altered level of gene repression by DNMT (68–70). Finally, enhanced activities of transcription factors such as hypoxia inducible factor 1 due to the high level of carbon monoxide and hypoxia have also been reported to influence airway gene expression (71).

Consequently, the combinatorial effect from both aberrant acetylation of histone and DNA methylation promotes the transformation of chromatin from a condensed structure to an activated open conformation. This facilitates irregular accessibility of DNA for transcription machineries, hence irregular gene expression by various cell types in the airway. The mechanisms reported are responsible for increased expression of NF-κB-dependent proinflammatory gene products [i.e., IL-1β, IL-6, IL-8, CCL-5 cyclooxygenase (COX)-2, and MIP-2/CXCL2] in both pulmonary structural cells (bronchial, small airway, and alveolar epithelial cells) and immune cells (alveolar macrophages), increased VEGF and iNOS in nasal fibroblasts and lymphocytes (Jurkat T cells), respectively, and decreased activity of antioxidant transcription factor Nrf2 and α1-antitrypsin in bronchial epithelial cells (54, 56, 57, 59, 62–64, 72–79). These may contribute to the anatomical anomalies in the airway and excessive inflammatory responses among smokers during the course of COPD.

### The inflammatory immune response in COPD

**Figure 2:**
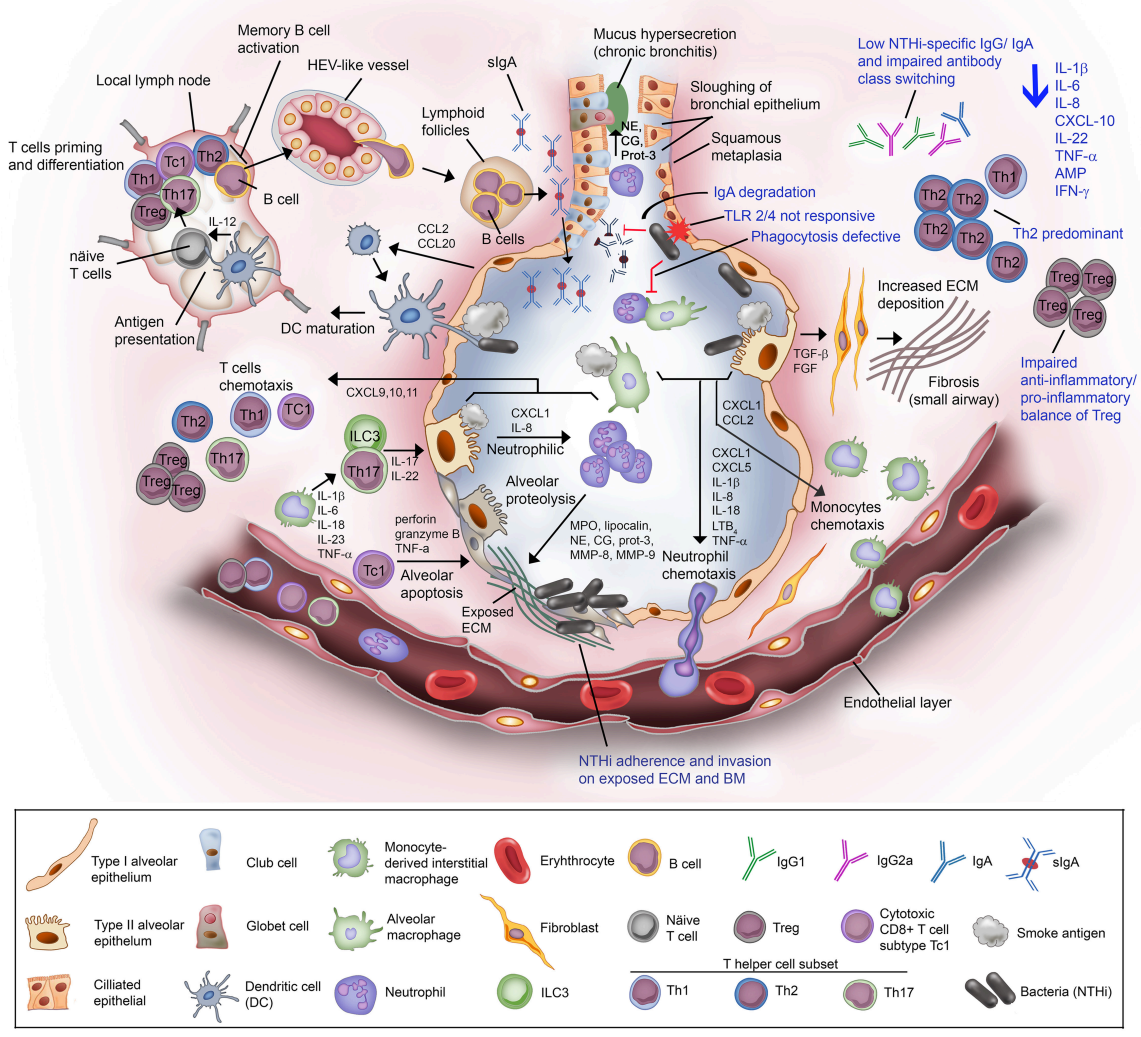
Non-typeable *H. influenzae*-dependent immune responses in the lower airway of COPD patients result in inflammation. Airway epithelium exposed to cigarette or tobacco smoke display an increased permeability with compromised tight junctions, and airway remodeling (goblet cell hyperplasia and small airway squamous metaplasia). Cigarette smoke causes the activation of airway epithelium and alveolar macrophages. The activated airway structural and resident immune cells release an array of chemotactic factors responsible for recruitment of inflammatory and immune cells to the lung. Activated epithelium produces TGF-β and FGF that triggers the production of ECM molecules by fibroblasts. Increased deposition of ECM causes progression of fibrosis and air flow limitation. The chemokines CXCL1 and IL-8, and LTB_4_ attract the circulating neutrophils through the receptors CXCR2 and BLT_1_, respectively. Meanwhile, CXCL1 and CCL2 targeting the receptors CXCR2 and CCR2 on blood monocytes are also released. Recruited blood monocytes differentiate into macrophages in the airway tissue. Activated alveolar macrophage and epithelium cell also release inflammasome (1L-1β and IL-18) for neutrophils survival and activation of helper T cells Th17. The chemokine IL-23 are released by macrophages to attract T helper cell subset Th17, and ILC3. Both Th17 and ILC3 will release IL-17 and IL-22 that will act on the alveolar epithelium to release CXCL1 and IL-8 for enhanced recruitment of neutrophils, resulting in neutrophilic inflammation. Activated neutrophils are thereafter degranulated and release myeloperoxidase (MPO), lipocalin, neutrophil elastase (NE), cathepsin-G (CG), proteinase-3 (Prot-3), and matrix metalloprotease (MMP) 8 and 9. The granulated products are proteolytic and elastilolytic to aveolar, causing alveolar destruction and emphysema. In addition, NE, CG, and Prot-3 are also targeting goblet cells and submucosal glands to induce hypersecretion of mucus. Dendritic cells carrying the receptors CCR2 and CCR6 are recruited to airway tissue via chemottractants CCL2 and CCL20. The dendritic cells uptake the antigen (smoke residues), and present the antigens to the naïve T cells at lymph nodes. Uncommitted T lymphocytes are thereafter primed to the presented antigen and activated by IL-12 derived from dendritic cells (professional antigen presenting cells; APC). Mature/activated T cells expressing receptor CXCR3 are chemotactic toward CXCL9, CXCL10, and CXCL11 and are recruited to the lung tissue. Cytotoxic CD8+ T cell subtype Tc1 releases perforin and granzyme B resulting in epithelial apoptosis contributing to emphysema progression. For the humoral immune response, B cells are activated by Th2, enter the circulation via high-endothelial venule (HEV)-like vessel and transported to lung tissue, and organized into lymphoid follicles at peripheral airway. B cell-derived plasma cells from lymphoid follicles release IgA, and secreted into airway lumen as secretory IgA (sIgA) via the polymeric immunoglobulin receptor. Mucosal antibodies play an important role to eradicate pathogens and noxious antigens via immune exclusion. However, the airway defense by sIgA is diminished by NTHi IgA protease that degrade the antibodies. TLR2 and TLR4 of the airway phagocytes and epithelium following exposure to cigarette smoke are not responding to P6 and LOS of NTHi. This results in defective phagocytosis and delayed bacterial clearance from the airway. The suppressed TLR4 induction in T cells has also lead to Th2 predominant immune response, with low production of IFN-γ and reduced T cell-mediated immune killing of NTHi. Moreover, NTHi downregulates Foxp3 of Tregs and thus impairs the anti-inflammatory/pro-inflammatory balance of Tregs. The extensive immunosuppressive activity by Tregs diminishes the response of effector T to NTHi stimulation. Lastly, plasma cells from COPD patients fail to produce NTHi-specific antibodies and compromised immunoglobulin class switching. The impairment of the host immune response in COPD toward NTHi infection are labeled in blue. In total, NTHi infection in COPD lung adversely reduces the production of IL-1β, IL-6, IL-8, CXCL-10, IL-22, TNF-α, antimicrobial peptide (AMP), and IFN-γ. This may explain the inefficient eradication of airway pathogens in COPD patients whereby persistent NTHi infection concomitantly escalates the inflammation and thus exacerbation in COPD.

COPD is associated with chronic inflammation in the peripheral airways orchestrated by both innate and adaptive immune responses that are interconnected via dendritic cells (80). Increasing numbers of inflammatory cells (neutrophils, macrophages, T and B lymphocytes, mast cell, eosinophils, and dendritic cells) and inflammatory mediators are accumulated in the airway lumen/wall in the lung parenchyma (19, 81). These immune cells and inflammatory mediators can hence be detected in the sputum and BAL fluid of COPD patients. The level of accumulation is positively correlated with disease severity. An increasing number of studies using animal models and clinical tissues have reported the nature of excessive airway inflammatory responses in COPD. Despite this, the heterogeneity in symptoms progression among COPD patients remain unexplained. The overall mechanism of COPD inflammatory immune response is depicted in Figure 2.

#### The first line of defense in the lung—the innate immunity and inflammasome

Lung structural cells (epithelial and endothelial cells, fibroblasts, and airway smooth muscle cells) are activated by inhaled irritants through the stimulation of several pattern recognitions receptors (PRRs), with Toll-like receptor (TLR)-4 being reported as the key player in most of the inflammatory responses (82–85). This causes an increased expression and release of an array of pro-inflammatory mediators and chemokines through the oxidative pathway by the activated bronchial epithelial cells and immune cells (alveolar macrophages). The inflammatory mediators [(interleukin (IL)-1β, IL-6, IL-8, IL-33, C-X-C motif chemokine ligand (CXCL) 10, granulocyte-macrophage colony-stimulating factor (GM-CSF), granulocyte-colony stimulating factor (G-CSF), tumor necrosis factor (TNF)-α, fibroblast growth factor 1 and 2 (FGF1/2), transforming growth factor (TGF)-β1, C-C motif chemokine ligand (CCL) 2, CCL20, and thymic stromal lymphopoietin (TSLP)] act on recruited immune cells and resident cells to initiate a series of innate immune responses (23, 86–90). Meanwhile, activated alveolar macrophages, which are usually patrolling the lung parenchyma, further release more pro-inflammatory mediators and chemokines [IL-1β, IL-6, IL-8, IL-23, TNF-α, CCL1, CXCL1, CXCL5 (ENA-78), CXCL9, CXCL10, CXCL11, CCL2, leukotriene B4 (LTB_4_)], ROS, elastolytic enzyme [matrix metalloprotease protein (MMP)-2,−9, and−12; and cathepsin-K,- L, and -S], GM-CSF, and G-CSF (23, 91, 92). The enhanced levels of CCL2 and CXCL1 result in recruitement of blood monocytes expressing CCR2 and CXCR2 (receptors for CCL2 and CXCL1, respectively), to the lung and differentiate locally into macrophages. Interestingly, there are higher expression levels of the CCR2 and CXCR2 found on blood monocytes in COPD subjects (93). This may explain the rapid recruitment and excessive accumulation of monocyte-derived interstitial macrophages in the lung tissue of COPD patients (94, 95).

Upregulation of neutrophil chemoattractors (LTB_4_, CXCL1, CXCL5, IL-8, and TNF-α) induces a massive migration of circulating neutrophils into the lung parenchyma (96). The transmigration of blood neutrophils occurs through adherence of the granulocytes to E-selectin of endothelial cells that is found to be upregulated in COPD (97). This results in airway neutrophilia in several COPD patients (96, 98, 99). The recruited neutrophils (to the lung) are then activated to secrete granule proteins [myeloperoxidase (MPO) and neutrophil lipocalin] while releasing its own IL-8 for further neutrophilic recruitment and amplification of the inflammation (100). In addition to the macrophage-derived proteases, neutrophils also secrete serine proteases [neutrophil elastase (NE), cathepsin G, proteinase-3, MMP-8, and MMP-9] that are associated with serious alveolar destruction in emphysema (101). The protease activity may be further enhanced in conditions with genetic deficiencies or suppressed expression of α1-antitrypsin by tobacco smoke. In addition, NE, cathepsin G, and proteinase-3 are involved in the stimulation of mucus secretion from submucosal glands and goblet cells, resulting in airway mucus hypersecretion and airway obstruction in COPD (101).

The NLRP3 (NLRP3: nucleotide-binding domain, leucine-rich-containing family, pyrin domain-containing-3 OR Nod-like receptor protein 3) inflammasome is a cytosolic multi-protein complex (consisting of the inflammation sensor protein NLRP3, adapter protein ASC, and the effector protein caspase-1) (102). The NLRP3 inflammasomes are involved in the COPD airway inflammation by regulating the production of pro-inflammatory cytokines IL-1α, IL-1β, and IL-18. These cytokines are important for neutrophil survival and activation of T helper (Th) 17 cells (103). Interestingly, local airway NLRP3 inflammasome activation is positively correlated with acute exacerbations and lower airway microbial colonization in COPD patients (103, 104). Moreover, in an elastase-induced emphysema model, the NLRP3 inflammasome is activated in addition to hyperproduction of mucin MUC5AC by diesel extract particles, extracellular ATP, and inflammatory protein S100 (105, 106).

#### The adaptive immunity in COPD

The adaptive immunity is initiated at a later stage, and is recognized by the increased number of T and B lymphocytes and pulmonary dendritic cells. Dendritic cells are the major antigen-presenting cells (APC) in the airways, and link the innate and adaptive immunity. Circulating dendritic cells (expressing receptors CCR2 and CCR6) are recruited to the airway via dendritic chemoattractants CCL2 and CCL20 released by activated airway epithelial cells in response to cigarette smoke (107, 108). Dendritic cells act by endocytosis of inhaled irritants that subsequently are processed into antigen peptides during maturation and further migration to lymph nodes.

Uncommitted T lymphocytes are thereafter primed by the presented antigen. These important cells are activated by IL-12 released from dendritic cells for subsequent commitment into antigen-specific T cell lineages, i.e., T helper 1 (Th1; CD3^+^CD4^+^) cells, whereas immature dendritic cells in the airway promote Th2 differentiation (23, 109). Interestingly, in COPD patients, pulmonary Th and cytotoxic T cells (Tc; CD3^+^CD8^+^) express more CXCR3 receptors compared to healthy individuals (110, 111). This enhances their migration toward chemoattractants CXCL9, CXCL10, and CXCL11 that are actively released by alveolar macrophages in COPD subjects. Activated CD8^+^ T cell subset type 1 (Tc1) releases perforins, granzyme B, and TNF-α to induce alveolar cells apoptosis, contributing to the emphysema (112). In parallel, pulmonary Th17 T cells are activated by alveolar macrophage-derived IL-6 and IL-23 to secrete IL-17A and IL-22 causing neutrophilic inflammation (113, 114). Inflammatory cytokines are also released by type 3 innate lymphoid cells (ILC3) (115). The ILCs are involved in the homeostasis of lung immunity and are regulated by epithelially produced IL-33 and TSLP (116, 117), and are further stimulated in response to cell damage.

The accumulation of B lymphocytes in the peripheral airway and within lymphoid follicles is associated with airway autoimmunity in the progression of COPD (118). Airway tissue damage in conjunction with impaired T-regulatory cells (Tregs), both related by cigarette smoke, contributes to the formation of autoantibodies against airway components. Autoantibodies against elastin, epithelial, endothelial, carbonylated, and citrullinated proteins are found in the circulation of COPD patients (119–124). The generation of autoantibodies might activate plasma exudate-derived complement components resulting in a chronic inflammation, and consequently damage of the airways with emphysema progression (124–127).

From a physiological point of view, a modulated inflammatory process is important for a protective and optimal immune response. However, the prolonged airway inflammation in COPD as a results of impaired homeostasis leads to serious side effects since it amplifies the tissue damage and impairs the local immune defenses. The abrogated local immune system may make the airways of COPD patients susceptible for opportunistic or recurrent infections by viruses and bacteria that in turn might exacerbate the disease.

## Acute exacerbations of COPD (AECOPD) and association with microbial colonization

Acute exacerbations of COPD (AECOPD) are episodes of acute symptom worsening that usually are associated with both respiratory (increased airway inflammation) and non-respiratory (system inflammation/co-morbidities) effects (128–130). The typical symptoms of an AECOPD include increased production of purulent sputum, dyspnea, cough, wheezing, and symptoms of a cold that may last from 7 days up to 12 weeks (15, 130, 131). It commonly occurs in patients with advanced COPD and results in additional therapy based on the level of exacerbations. Exacerbations are classified in three levels according to GOLD. There is the mild disease that can be treated with short acting bronchodilators (SAB); moderate disease with SAB combined with antibiotics and/ or oral corticosteroids; and finally severe exacerbations with acute respiratory failure which requires emergency room visit and eventually hospitalization (15, 130).

AECOPD is a complex yet multifactorial consequence of COPD. Most of the exacerbations could be triggered by infectious (up to 80%) or non-infectious agents (~10%) (AECOPD with known etiology), whereas up to 30% of cases are of unknown etiology (132, 133). Respiratory tract infections are the major causes for AECOPD with known etiology and are mainly attributed to infections by viruses, bacteria, and atypical bacteria (not detected with conventional Gram-staining) (11, 134, 135). Non-infectious causes of AECOPD include air pollution, environmental factors, meteorological effects, and comorbidities of the patients, all of which are partially contributing to COPD exacerbations (133, 135, 136).

### Viral and bacterial infections in AECOPD

Respiratory viral infections are often the primary cause in the infection-dependent AECOPD, and virus was identified as single or multiple infecting strains from up to 64% of COPD patients with exacerbations recorded between years 2001–2017 (137–145). The most common infecting viruses are, by far, human rhinovirus, influenza virus A, and respiratory syncytial virus, whereas parainfluenza virus, coronavirus, echovirus, human metapneumovirus, and adenovirus are considerably rare.

Bacterial infections contribute to an average of 50% of infective acute exacerbations with a prevalence being reported ranging from 26 to 81% (132, 135, 146–148). The most commonly pathogenic bacterial species isolated from the lower airway of COPD patients during AECOPD are NTHi, *Moraxella catarrhalis, Streptococcus pneumoniae, Staphylococcus aureus, Pseudomonas aeruginosa*, and *Klebsiella pneumonia*e (11, 129, 133, 136, 149–152). It has been suggested that infection with new strains of the infecting species, rather than a new species, is highly associated with an increased risk of exacerbation (11, 153, 154). Atypical bacteria that cause exacerbations are *Chlamydia* spp.*, Legionella pneumophilia*, and *Mycoplasma* spp.

In contrast to viral infections that are diagnosed in 5–45% of COPD patients with stable disease and increase to 39.3–64% during COPD exacerbations, bacterial colonization in the airways are more common with the same species during both stable disease (25–86%) and exacerbations (58.8–81%) (11, 132, 136, 137, 142, 155–158). Hence the precise or direct role of bacterial infection as the primary cause in triggering AECOPD remains controversial although a significantly increased bacterial load is observed during exacerbation in several patients. This further suggests that bacteria might be more involved as secondary invaders after an initial viral infection.

Viral infections have been reported to cause several physiological changes in the lung that in turn facilitates secondary bacterial invasion. The mechanism of bacterial superinfection has been described for *H. influenzae, S. pneumoniae, S. aureus*, and many other airway pathogens (159–161). Firstly, viral infections destroy the tight junctions of the airway epithelial barrier while inducing epithelium apoptosis. This results in the onset of airway epithelium lining repair whereby the sloughed off dead cells would become a rich nutrient source for growth of infecting bacteria. The damaged epithelium lining also enables bacterial adherence to the exposed basement membrane and ECM. Secondly, the demolished ciliated clearance as a result of the virus-damaged airway epithelium lining further promotes bacterial colonization and subsequent epithelial transmigration into deeper tissues (162–164). Lastly, viral infections are also detrimental to the airway immune defense by causing degradation of antimicrobial peptides (AMP), and by triggering IFN-γ secretion by immune cells. This results in suppressed macrophage and neutrophil responses to infecting bacteria, and thus enables bacterial evasion of the airway immune defense (165–168). Nevertheless, viral and bacterial coinfection have greater impact in the AECOPD airway inflammatory responses than bacteria or virus infection alone (168, 169). This is in parallel with the co-isolation of both respiratory viruses and bacteria from 6 to 30% of AECOPD patients (129, 133, 136, 170–174).

Infective AECOPD is also attributed to impaired functions of AMP, macrophages, and neutrophils triggered by inhaled irritants such as tobacco smoke. Expression of microbial-induced AMP (human β-defensin 2) is suppressed in airway epithelial cells when exposed to cigarette smoke (175, 176). Both the alveolar and monocyte-derived macrophages in patients with COPD are defective in phagocytosis of bacteria such as *H. influenzae* and *S. pneumoniae* (177, 178), and in efferocytosis of apoptotic neutrophils and epithelial cells. In addition, neutrophils from COPD patients are aberrant in chemotactic response with defective accuracy (179). All these factors contribute to the failure to resolve inflammation in COPD leading to facilitated chronic microbial colonization, also during exacerbations.

### The role of the lung microbiome in AECOPD

The low number of cultivable bacteria found in healthy individuals previously led to the conclusion that healthy and normal lungs are virtually sterile. This hypothesis is currently being revised, since the introduction of 16S rDNA based molecular diagnostics has shown that even healthy lungs have a distinct microbial community, different from that seen in the upper respiratory tract (180, 181). This has led to the concept of a core human lung microbiome which can be altered in COPD stable disease and during exacerbations (182). The role of the lung microbiome in the pathogenesis of COPD by influencing host immune response has also been suggested (151, 183–188).

The stability of the lung microbiome has profound impact on maintaining local immune homeostasis (189). According to the “vicious circle” hypothesis, airway inflammation and impaired immune defenses caused by either viral infections or irritant inhalation have ecological influence on the airway microenvironment and growth conditions that would eventually lead to dysbiosis of the lung microbiota (182, 190). The changed lung microbiome would then cause a maladaptive immunological response resulting in further inflammation and damage of the lung immune defenses, and additional alteration of the lung microbiome. The chain of events thus generates a vicious circle that contributes to COPD progression and exacerbation.

Several studies have documented that COPD progression from stable state to an exacerbation could induce microbiota shift in the lower airway (bronchioles), sputum, and throat (151, 190–196). Alteration in the microbiome complexity or richness is associated with the inflammatory process and changes in ECM protein expression in the lung, as observed in COPD (185, 197). Declined diversity in the lung microbiome has been reported to be related to disease severity, inflammation and decreased lung functions in COPD. This includes the increased emphysematous destruction, bronchial tissue remodeling, lymphoid follicle formation, elevated autoantibodies, and IL-17A production, and finally increased neutrophil extracellular traps (NET) formation in the airway of animal models or AECOPD patients (198–201). It has recently been reported that lung microbiome diversity is also associated with genetic factors. Mannose-binding lectin (MBL) deficiency has also been associated with disease severity and exacerbations in patients with cystic fibrosis and bronchiectasis (202). However, COPD patients with a genetic deficiency in MBL are less susceptible to *Haemophilus* spp. colonization, lowering the risk of exacerbations while their lung microbiota is more diverse than normal COPD patients (203).

## The clinical role of NTHi in COPD

In this review we will focus on NTHi, one of the dominant genera that is relatively abundant in the total COPD-dependent lung microbiome, due to its role of infection in COPD immunological responses (136, 149, 192–195, 198, 204–206).

The microbiology of *H. influenzae* has recently been reviewed in detail by our group and others (4, 5, 207). It is a Gram-negative coccobacillus that commonly colonizes the human nasopharynx, and is typed as capsulated (type a–f) or non-encapsulated strains (NTHi). *H. influenzae* may cause both invasive and mucosal disease (208). Since the introduction of capsule polysaccharide conjugate vaccines against type b (Hib), NTHi dominate, followed by capsule type f (Hif) (209, 210). Mucosal infections, including acute otitis media, sinusitis, and exacerbations in COPD, are nowadays mainly associated with NTHi. There has also been a significant shift in the epidemiology of severe invasive disease, from Hib infections in small children to NTHi in adults (210, 211). The most common principal infection focus by *H. influenzae* is now community acquired pneumonia (CAP), whereas the incidence of historically common diagnoses such as meningitis and epiglottitis have significantly decreased (210, 211). Patients with underlying conditions, notably COPD, seem to be at higher risk for invasive infections (209).

There is consensus that *H. influenzae* is one of the key bacterial pathogens involved in pathogenesis of both stable COPD disease and acute exacerbations (207). However, the relative abundance and significance of NTHi in COPD varies between different studies. Several factors, such as sampling methodology, choice of microbiological analysis and, if the patient has a stable disease or an exacerbation, or has been subject to previous antibiotic therapies, tend to affect the outcome of the studies (212).

Common sampling methods from the lower respiratory tract include both bronchoscopy techniques such as protected specimen brush (PSB) and collection of BAL fluid as well as non-invasive methods like sputum sampling (213). All of these methods, particularly sputum, are to some extent subject to the risk of contamination from the normal microbial flora of the oro- and nasopharynx, which might reduce their specificity (213). However, several studies still show a distinct association between lower respiratory tract samples and clinical parameters in COPD patients, making the information valuable (214).

Cultivable bacteria are seldom found in the lower airways of healthy individuals (215), whereas COPD patients show bacterial growth in 30–50% of cases even during stable disease (Table 1). On top of that, several studies have shown a significant increase in the *Proteobacteria* phylum, which includes *Haemophilus* spp., in individuals with both stable disease and AECOPD (Table 2). NTHi is consistently one of the predominating bacterial species isolated in those cultures; other important pathogens include *S. pneumoniae, M. catarrhalis*, and *P. aeruginosa* (219). During AECOPD, the bacterial load is increased even further, and NTHi continues to be the predominating species (208). Furthermore, acquisition of a new NTHi strain has, in one study, been linked to the onset of AECOPD (153). Moreover, the growth and dominance of *H. influenzae* following rhinovirus infection was observed in the sputum microbiome of patients with COPD (190).

**Table 1:** Abundance and significance of NTHi and other potentially pathogenic bacteria in healthy individuals and various stages of COPD using culture-based methods.

**Sample type**	**Microbiological analysis**	**Main findings**			**Study**
		**Healthy individuals**	**AECOPD^b^**	**Stable COPD disease**
PSB^a^	Quantitative culture	Potentially pathogenic bacteria found in 4% of patients	Potentially pathogenic bacteria found in 54% of patients	Potentially pathogenic bacteria found in 29% of patients	Rosell et al. (148)
		NTHi found in 3% of patients	NTHi found in 30% of patients (predominating species)	NTHi found in 17% of patients (predominating species)
BAL^c^	Quantitative culture	Potentially pathogenic bacteria found in 0% of ex-smokers and in 20% of non-smokers (no NTHi)	N.A.^d^	Potentially pathogenic bacteria found in 34% of patients	Sethi et al. (216)
				NTHi predominating species
Sputum/Throat swab/PSB /BAL	Culture and molecular typing of strains	NTHi recovered in 35% of patients at any respiratory site	NTHi recovered in 7% of intubated patients with respiratory exacerbation	NTHi recovered in 57% of patients at any respiratory site	Bandi et al (217)
Sputum	Culture and molecular typing of strains	N.A.	Isolation of a new strain of NTHi significantly associated with exacerbation	N.A.	Sethi et al. (153)
			Patients with a new NTHi-strain twice as likely to have AECOPD	
Sputum	Quantitative culture	N.A.	Potentially pathogenic bacteria found in 70% of exacerbations	Potentially pathogenic bacteria found in 48% of patients	Wilkinson et al. (172)
			NTHi predominating species (38%)	NTHi predominating species (14%)
Sputum	Quantitative culture	N.A.	Potentially pathogenic bacteria found in 55% of exacerbations	Potentially pathogenic bacteria found in 38% of cultures	Papi et al. (171)
			NTHi predominating species	NTHi predominating species

a *PSB, protected specimen brush*.

b *AECOPD, acute exacerbation of COPD*.

c *BAL, bronchoalveolar lavage*.

d * N.A., not applicable*.

**Table 2:** Abundance and significance of NTHi and other potentially pathogenic bacteria in healthy individuals and various stages of COPD using molecular methods.

**Sample**	**Microbiological analysis**	**Main findings**			**Study**
		**Healthy individuals**	**Stable COPD disease**	**AECOPD**
Sputum	16S rDNA	N.A.	Baseline^a^	Significant increase of the Proteobacteria phylum (which includes *Haemophilus* spp.)	Huang et al. (195)
Sputum	16S rDNA	N.A.	Baseline^a^	An increase in relative abundance of *Haemophilus* spp. as well as other bacteria typically associated with exacerbations	Millares et al. (218)
Sputum	16S rDNA	No increase in numbers of proteobacterial sequences following rhinovirus infection	N.A.	Significant increase in numbers of proteobacterial sequences, mainly *H. influenzae*, following Rhinovirus infection	Molyneaux et al. (190)
Sputum	16S rDNA	N.A.	Baseline^a^	An increase in the relative abundance of *Haemophilus* spp. following exacerbation, an increase of *Haemophilus* following corticosteroid treatment but a decrease after antibiotic treatment	Wang et al. (149)
Sputum	16S rDNA	N.A.	A significant increase in the abundance of *Haemophilus* with increasing disease severity	No significant increase in abundance of *Haemophilus* genera during exacerbation	Mayhew et al. (151)

a *Comparison of the microbiota sampled from a patient during stable disease (defined as baseline) and during an exacerbation*.

## Colonization and adaptation of NTHi in the lower airways of COPD patients

The chronic inflammation that characterizes COPD pathogenesis causes significant changes to the pulmonary tissue. The lower respiratory tract of patients suffering from this disease is marked by epithelial denuding, hypersecretion of mucus, disproportionate phagocyte presence and imbalances in antioxidant/oxidants (220). This altered *milieu* selects for specific bacterial species that are genetically equipped to competently address these environmental stressors (151, 195, 221). NTHi is the most common pathogen isolated from the sputum of COPD patients, and the primary cause of exacerbations (212), indicating a unique ability to colonize and persist in the chronically inflamed lower respiratory tract.

In recent years, great efforts have been made in understanding how NTHi colonizes the pulmonary tissue. In addition to the regular arsenal of virulence factors associated with NTHi (5), the bacterial pathogen undergoes specific adaptations to increase its fitness in the COPD setting. Specific genetic islands that include *ureABCEFGH, lic2b, hgbA, iga, hmw1*, and *hmw2* have been reported to be enriched in NTHi strains isolated from COPD patients compared to commensal NTHi (222). These genes are involved in raising the pH of the environment, lipooligosaccharide (LOS) synthesis, iron uptake, immune evasion, and attachment to host tissue. The validity of these findings is strengthened by previous work identifying upregulation of many of the same bacterial gene products during growth in COPD sputum (223). Moreover, peroxiredoxin-thioredoxin, an antioxidant enzyme, was found to be one of the most enriched proteins in NTHi during growth in COPD sputum, suggesting that the bacteria upregulate oxidative stress-countermeasures when facing oxidative imbalances in the diseased lung (223). Oxidative stress resistance has previously been shown to be vital for NTHi survival in infection models (224).

In a seminal investigation by Pettigrew et al., whole-genome sequencing (WGS) was conducted to follow the *in vivo* adaptation of NTHi to the COPD environment over time (225). Several interesting findings were reported in this work. Firstly, the median duration of persistence by the pathogen was found to be 161 days, but it could persist in patients for up to as many as 1,422 days. Secondly, slipped-strand mispairing-mediated phase variation was identified as the primary genetic adaptation to the niche. Poignantly, the genes affected by the regulation mechanism encoded for (among others) the HMW adhesins, LOS biosynthesis, and iron uptake, that is, the same processes identified in the previous studies as important for COPD adaptation (222, 223). Thirdly, and somewhat surprising, it was observed that a very limited number of genes were gained/lost during persistent colonization, meaning that selection for strains that thrive in the inflamed lower airways occurs at the very onset of colonization. Finally, the authors reported that genetic changes occurred in 8 of the 12 investigated vaccine antigens during persistent infections, a fact that might be taken into consideration for potential vaccine development against NTHi.

Another virulence factor that has been reported by Murphy and co-workers to play a pivotal role for NTHi survival in COPD settings is IgA-protease, a hydrolytic enzyme that cleaves secretory IgA (sIgA) antibodies in the mucosal epithelium (226–228). Four genes encode for the same number of different variants of the endopeptidase with various cleavage site specificities: two *igaA* (*igaA1* and *igaA2*) and two *igaB* (*igaB1* and *igaB2*). The *igaA* is present in all NTHi whereas *igaB* is present in ~40% of the strains (226). The *igaB1* gene has been reported to be more prevalent in COPD exacerbation-causing strains, although the *in vivo* expression levels did not differ from asymptomatic colonization strains that also carried the gene (226). However, IgA-protease B1 and B2 have been found to promote the intracellular survival of NTHi in human epithelial cells, providing a secondary function (in addition to hydrolysis of IgA antibodies) that could facilitate NTHi growth in inflamed environments (227). While a majority of the persistent NTHi strains that dwell in COPD patients continuously express one or more variants of the enzyme, it has recently been found that a phase variation to an OFF-state can occur via slipped-strand mispairing over time (228). This suggests that during certain conditions, there is a fitness benefit in not expressing *iga* in the airways of COPD patients, albeit the specifics of this process are currently unknown.

Another interesting aspect of NTHi colonization of COPD patients is with regard to biofilm formation (229). NTHi strains that colonize the Eustachian tube causing otitis media are known to build up biofilms *in situ* (230). However, strains isolated from COPD patients tend to have significantly diminished ability to form biofilm compared to invasive strains or those isolated from otitis media patients (229), suggesting that this mechanism is not important for survival in the COPD niche. As biofilms tend to protect the bacterial community from external assaults, these findings could indicate that the hypermucoid milieu in the COPD airways is severely impaired in its ability to deliver an apt immune response for optimal clearance of residing microorganisms. In light of this impairment, biofilm formation might not be necessary for NTHi to persist in this particular environment.

Infections with NTHi have also been shown to reduce cellular levels of E-cadherin, a protein required for tight junction formation and epithelial cell integrity in human cells (231). Considering that perturbations in the epithelial cell barrier caused by the loss of E-cadherin is a common symptom of COPD, NTHi-mediated exacerbations likely contribute to this step of COPD pathogenesis. The subsequent denuding of the epithelium could facilitate microbial colonization of the basal lamina, a well-established virulence mechanism employed by NTHi and other pathogens (232). It is currently unknown which bacterial virulence factor(s) that induce the reduction of E-cadherin levels in the host.

In summary, investigations from recent years show that the environment of the lower respiratory tract of COPD patients selects for NTHi strains that can upregulate adhesins, modify LOS biosynthesis pathways, increase antioxidant stress responses and cellular invasion strategies, and, finally, trigger tolerance against acidic pH. These important colonization mechanisms thus provide researchers with viable targets for developing novel therapies.

## NTHi-dependent airway immune responses in COPD

NTHi is a commensal in the nasopharyngeal site but is often associated with strong inflammatory responses in the lower respiratory airways, especially in patients with COPD, bronchiectasis, cystic fibrosis, pneumonia, or idiopathic pulmonary fibrosis (11, 233). Colonization and subsequent infection of NTHi in the lower airways of COPD patients elicits episodes of immune responses orchestrated by both the innate and adaptive immunity. NTHi infection is thus commonly associated with inflammation that is mainly mediated by transcription factor NF-κB-dependent production of proinflammatory mediators. The activation of NF-κB requires induction of cross-signaling networks and cascades via activation of PRRs (pattern recognition receptors) of host innate immune cells (234). Unresolved or prolonged (chronic) inflammation or failure to restore the homeostatic inflammatory status potentially contributes to exacerbations. This is clearly shown in murine COPD simulation models with NTHi-triggered inflammation (235–237). Mice exposed to NTHi lysates display inflamed airways loaded with increased levels of inflammatory mediators and phagocyte infiltrates. Moreover, multiple exposures to bacterial lysates which may represent a chronic NTHi infection caused extremely high infiltration of phagocytes and lymphocytes in the airways of this particular mouse model. In addition, the airway walls of the infected animals were also thickened due to increased collagen deposition (fibrosis) that reflects the typical COPD features. The host immune response and specific interactions during NTHi infection in COPD is summarized in Figure 2.

### NTHi stimulation of PRRs in immune activation

The epithelium and alveolar macrophages are predominant cell types in the airway compartment. They comprise the first line of defense in the cellular immune response against potential inhaled pathogens and antigens. The sensing of bacteria, and particularly NTHi in the lower airways is initiated via PRRs expressed on innate immune cells and endothelium in addition to epithelial cells (238–240). TLRs are PRRs that sense stimulation by NTHi-derived pathogen-associated molecular patterns (PAMPs), and play a primary role in initiating effector cellular responses and intracellular signaling for NF-κB activation (238). Among the different TLRs, most of the studies on NTHi infection have by far been focused on TLR2 and 4. Lipoproteins including NTHi P6, and LOS are potent immunomodulators for activation of TLR2 and TLR4, respectively, and has been described in several studies on airway epithelial cells and alveolar macrophages (241–244).

Interaction of NTHi lipoprotein P6 with TLR2 on human epithelial cells [type II alveolar A549 and human middle ear epithelial cells (HMEE)] causes NF-κB-dependent activation via two distinct TLR-signaling pathways, that is, the NF-κB translocation-dependent, and -independent pathways (242). The NF-κB nuclear translocation-dependent pathway requires activation of NF-κB-inducing kinase IKK complex. In the second pathway, the MKK3/6-p38 MAPK signaling cascade is recruited for direct nuclear phosphorylation, and thus activation of NF-κB. The branching of both pathways may occur at the TGF-β activated kinase 1 (TAK1) signaling junction. NTHi stimulation via TLR2 and downstream activation of p38 MAPK/NF-κB-dependent pathways result in expression of COX-2 and prostaglandin (E2) (PGE2) that promote inflammatory responses (245).

TLR4 stimulation by NTHi LOS also contributes to the activation of NF-κB via two signaling pathways, the primary activating pathway of MyD88 cascade and the alternative pathway of Toll/IL-1R domain-containing adapter-inducing interferon-β (TRIF). Both pathways activate NF-κB through phosphorylation and degradation of inhibitor IκBα (243, 246). NTHi-TLR4 signaling mediates an effective innate immune response that leads to upregulation of TNF-α, IL-1β, IL-6, macrophage-inflammatory protein (MIP)-1α, MIP-2, and neutrophil infiltration in the airways of mice. The TLR4 response promotes efficient pulmonary clearance of bacteria in TLR4-expressing animals compared to CD14/TLR4 knockout mice (243, 244). A recent study by Jungnickel et al. revealed that, in parallel with the infection-induced pulmonary neutrophilic inflammation, NTHi-dependent stimulation of both TLR2 and TLR4 in a transgenic mouse [(Kras^LA1^) with oncogenic Kras allele in the lung epithelium] additionally promotes the proliferation of Kras-induced early adenomatous lesion in the lung in an TLR-dependent manner (247). The association or role of NTHi-induced airway inflammation in lung cancer progression, however, is not supported by another recent cohort study showing the lack of differences in NTHi specific-antibodies between cancer- and non-cancer COPD patients (12).

Lastly, Dectin-1 and the epidermal growth factor receptor (EFGR) pathway also have proinflammtory effects upon interaction with NTHi (248, 249). Activation of the Dectin-dependent proinflammatory response requires NTHi-induced phosphorylation of the Dectin-1 hem-immunoreceptor tyrosine-based activation motif (hemITAM) (248). Direct activation of EFGR in alveolar cells and HMEE by NTHi-derived EGF-like factor has been shown to contribute to NF-κB activation. The EFGR-dependent NF-κB activation is mediated via an NF-κB nuclear translocation-independent pathway, which involves both MKK3/6-p38 and PI3K/Akt signaling pathways (249). Surprisingly, the interaction of EFGR and NTHi also results in negative regulation and suppression of the induction of TLR2 via the Src-MKK3/6-p38 α/β MAP kinase-dependent signaling cascade, and this in turn may facilitate NTHi infection (250). The actual components of NTHi that exhibit the EGF-like factor activity have, however, yet to be defined. The EFGR-dependent negative regulation of TLR2 may thus suggest a novel mechanism targeted by NTHi for immune evasion by attenuating the responses of host PRR, despite the contradicted role of EFGR in proinflammatory and innate immune responses of the airway epithelium (251). NTHi infection also upregulates the NRLP3-inflammasome during NTHi-induced inflammation in the airway epithelium and alveolar macrophages, leading to increased secretion of IL-1β and IL-8, and thus neutrophilic influx to the lung (252).

### Synergetic action of NTHi and inflammatory mediators

Some of the endogenous inflammatory mediators that are produced in response to NTHi infection, including TNF-α, IL-1α, and TGF-β1, may act synergetically with NTHi on the airway epithelial and immune cells. The synergetic interaction drives a positive feedback loop to amplify the NF-κB transcriptional activity on proinflammatory genes and further augments airway inflammation.

The synergetic activation of NF-κB by NTHi and TNF-α in HMEE and normal human bronchial epithelial (NHBE) cells occurs via NF-κB nuclear translocation-dependent and independent pathways. The latter pathway involves MAPK/extracellular signal regulated kinase kinase kinase 1 (MEKK1)-dependent activation of MAPK kinase 3/6–p38 MAPK pathway (253). However, the synergetic action of NTHi with TGF-β1 is mediated by another mechanism which involves Smad3/4-protein kinase A (PKA)-p300-dependent signaling cascade. The pathway components, PKA and p300, phosphorylates residue Ser276 and acetylates Lys221 of the NF-κB subunit p65, respectively. This results in enhanced DNA-binding activity of NF-κB (254).

The synergetic action of NTHi with both TNF-α and TGF-β1 enhances the production of TNF-α, IL-1β, and IL-8 from airway epithelial cells and interstitial polymorphonuclear infiltrates. Recently, it has been reported that co-infection of human rhinovirus and NTHi on the airway epithelial cells (NHBE cells and the BEAS-2B cell line) also results in synergetic induction of CCL20 and IL-8, albeit the exact mechanism remains to be elucidated (255). Of note, activated macrophages also release increased concentrations of TNF-α and IL-1α (256), further enhancing the inflammatory synergetic effect of surrounding immune cells.

Finally, IL-1α acts synergetically with NTHi to upregulate the expression of AMP β-defensin 2 (DEFB-4) via the p38/MAPK pathway (257). Of note, IL-1α could also act individually to upregulate the expression of DEFB-4 via the Src-dependent MEK1/2-ERK1/2 signaling pathway (258). Taken together, the synergetic action may aid in the expansion of the inflammatory response and in some cases worsen the clinical outcome.

### Phagocytosis of NTHi by airway phagocytes

Alveolar macrophages located in the air-parenchyma interface are the primary professional phagocytes in the lung (259, 260). These cells are responsible for infection eradication through its phagolysosomal machinery while releasing a plethora of inflammatory cytokines and chemokines for promoting a local inflammatory response and recruitment of neutrophils. Neutrophils are the first responder cells recruited from circulation to the airway for efficient killing of pathogens through an array of microbicidal strategies (261, 262). During NTHi lung infection, both alveolar macrophages and neutrophils are the main innate immune cells involved in the pulmonary bacterial clearance through phagocytosis. They are also an important source of cytokine secretion required for induction of other immune cells and enhanced bacterial killing. Eradication of NTHi by alveolar macrophages involves adhesion or contact, phagocytosis and phagolysosomal processing of bacteria, in addition to secretion of TNF-α. Phagocytic clearance of NTHi by alveolar macrophages is orchestrated through actin polymerization, plasma membrane lipid rafts, and phosphatidylinositol 3-kinase (PI3K) signaling cascade upon induction of macrophage PRRs by NTHi (256).

Interestingly, in response to NTHi infection, human alveolar macrophages, and blood neutrophils produce extensive amount of intracellular and extracellular ROS as a component of the antimicrobial defense. This leads to the formation of macrophage and neutrophil extracellular traps (METs and NETs, respectively), with co-expression of MMP-12 for enhanced bacterial killing (263, 264). Nevertheless, the overexpression of MMPs may adversely result in a protease imbalance and contribute to alveolar emphysematous destruction and bronchiectasis in COPD (265). Moreover, excessive endogenous ROS production could also introduce airway oxidative stress that is detrimental by causing chronic inflammation and tissue damage in the lung, and thus contributing to the COPD exacerbation (266, 267). The NET formation is elicited mainly by NTHi LOS in addition to other *Haemophilus* PAMPs (264).

### Cellular and humoral immunity in NTHi evasion

Several studies by King et al. have revealed that T cell-mediated adaptive immune responses against NTHi airway infection in patients with idiopathic bronchiectasis and COPD has been predominated by a Th2/Tc2 response (268–270). The activated T cells produce reduced level of the CD40 ligand and IFN-γ, and increased levels of TNF-α, IL-13, and IL-17, as well as altered IgG subclass production by plasma cells. It is to be noted that the Th2/Tc2-mediated immune response is less effective in suppressing NTHi infection. Redirecting the Th2/Tc2-mediated immune response to Th1/Tc1 dominant (which is more protective) by adding the Th1/Tc1 mediators (CD40 ligand and IFN-γ) has helped to restore the T cell-mediated immune killing of NTHi (269). However, a separate study in a COPD mouse model by Lu *et al*. reported that NTHi infection causes increased production of airway type 1 interferon (1-IFN) (271). It was further reported that DNA of NTHi acts as a PAMP in stimulating the STING/TBK1/IRF3 pathway, and thus the production of 1-IFN. The impact of the bacterial DNA-induced 1-IFN in host immune/inflammatory response, which may potentially induce a Th1/Tc1 response requires further investigations.

COPD patients also have abnormally higher number of Treg cells, myeloid-derived suppressor cells (MDSC), and exhausted effector T cells (PD-1^+^) than healthy individuals (272, 273). Cigarette smoke-induced anti-inflammatory activity of Tregs in a COPD model is further suppressed by NTHi infection. The pathogen causes downregulation of Foxp3 (biomarker of Tregs), and thus impairs the anti-inflammatory/pro-inflammatory balance of Tregs (273, 274). This may lead to the extensive immunosuppressive activity by Tregs on the proliferation of NTHi P6-specific effector T cells, causing a diminished response of effector T cells to sputum IL-6 and IL-8 induction, and increased levels of IL-10 and TGF-β1 (272, 275). Recently, it has been reported that mucosal-associated invariant T cells (MAIT) from COPD patients are more effective in response to NTHi stimulation and thus produce increased levels of IFN-γ, 3-, to 10-fold more than the COPD Th (CD4^+^) and Tc (CD8^+^) cells (276). However, the pulmonary MAIT cell immune responses are compromised in the presence of corticosteroids that are commonly used for the treatment of COPD. This may potentially prone the T cell-mediated immunity to a Th2/Tc2 response in COPD patients treated with corticosteroids (277). Interestingly, antigen-specific Th17 cells from NTHi-immunized non-COPD mice model recognize both homologous and heterologous strains of NTHi, and are able to confer protection upon adoptive transfer (278). However, it is unclear whether the Th17 cell which is prone to the inflammatory response could be “trained” to counteract the NTHi infection in COPD patients, particularly during exacerbations.

During the systemic humoral immune response in NTHi-infected COPD patients, greater concentrations of NTHi-specific IgG, IgA, IgM, and IgE serum antibodies are produced compared to non-infected controls (12, 279–281). Some of the NTHi-specific serum immunoglobulins are specific to P2, P5, and P6 (12, 282, 283). However, decreased mucosal antibodies associated with sIgA deficiency, or decreased total IgG in the small airways have been reported in COPD patients, and might be associated with disease severity (283, 284). Importantly, NTHi-specific mucosal sIgA has been found to be lower in the airways of NTHi-infected COPD patients than the non-colonized patients (285, 286).

The epithelial polymeric immunoglobulin receptor (pIgR) is essential for the generation of mucosal sIgA. It is, however, downregulated in COPD patients with a positive correlation to disease severity and increased level of TGF-β (287). The combinatorial effects of downregulated *plg*R and elevated TGF-β1 contribute to an impaired mucosal IgA immunity in COPD patients. A mouse model lacking the *pIg*R (^−/−^) is therefore devoid of sIgA and are susceptible to airway stimulation by an NTHi lysate resulting in increased inflammation and airway neutrophilia. Interestingly, introduction of exogenously added sIgA mitigated the airway inflammation (288). NTHi-infected COPD patients with greater airway inflammation have also decreased NTHi-specific mucosal IgG1 in the BAL fluid compared to the non-colonized patients (283). Interestingly, the phenomenon with decreased NTHi-specific antibodies seems to be restricted to the airways, since the specific serum antibodies are not affected. Therefore, the reduced mucosal IgG is unlikely to be associated with hypogammaglobulinemia (IgG deficiency), despite the latter was reported as a contributing factor in NTHi infection (289). Decreased airway IgA might be attributed to the expression of IgA proteases by NTHi. The bacterial IgA protease degrades the local airway IgA during airway colonization to avoid immune exclusion by sIgA (226, 228). Reduced mucosal antibodies might promote host immune evasion and resistance to complement-mediated killing of NTHi, thus enable persistent colonization of NTHi in the airways of COPD patients, in addition to a plethora of various other virulence mechanisms (4, 5, 207).

## Impaired immunity in COPD in response to NTHi infection—currently known mechanisms

In a cohort study of stable COPD patients, augmented airway inflammation and plasma fibrinogen, but not systemic inflammation, were found to be constantly correlated with the increased bacterial load (233). Higher numbers of NTHi has a greater impact than *S. pneumoniae* and *M. catarrhalis* in triggering inflammatory responses as measured by the augmented levels of inflammatory cytokines in sputum including IL-8, MPO, and 1L-1β. The increased inflammatory response in affected patients is potentially attributed to the persistent colonization of NTHi in the lower airway (207, 233). The compromised innate immune response in COPD, particularly the decreased microbicidal activity, has been regarded as one of the culprits for persistent airway colonization by NTHi, and is highly associated with COPD exacerbations (Figure 2).

### TLR tolerance: unresponsive to NTHi antigen stimulation

Whilst the role of macrophage extracellular traps (MET) for killing of NTHi remains unknown, it has been reported that blood neutrophils and NET from COPD patients are defective in the killing of planktonic or biofilm/NET-entrapped NTHi, respectively (263, 264, 290). A series of studies by Berenson et al. revealed that alveolar macrophages derived from COPD patients are basically dysfunctional in eradication of NTHi (177, 291–293). Intriguingly, TLR2 and TLR4 expressed on alveolar macrophages from COPD patients are intrinsically unresponsive to the potent immunomodulatory lipoprotein P6 and LOS, respectively. This causes decreased LOS/P6-induced expression of TLRs, reduced NF-κB nuclear activation and consequently diminished IL-8, TNF-α, and IL-1β responses by alveolar macrophages from COPD patients. The compromised TLR expression and signaling potentially contribute to the defective complement-dependent and independent phagocytosis of NTHi. The defective phagocytosis is greater for NTHi than for *M. catarrhalis*, and correlates with disease severity. Interestingly, the phagocytosis disability was not detected in monocyte-derived macrophages in COPD. In contrast, however, Taylor et al. reported that monocyte-derived macrophages from COPD patients are also defective in phagocytosis of NTHi and *S. pneumoniae*. The author also suggested that the defective monocyte-derived macrophages are not attributed to the alteration in cell surface TLR2 or TLR4 expression, macrophage receptor with collagenous structure (MARCO), CD163, CD36 or the mannose receptor (178). The unresponsive TLR2 and TLR4 in COPD alveolar macrophages to NTHi lipoprotein and LOS might be explained by the recently reported phenomenon of TLR tolerance (294). Repetitive stimulation of COPD alveolar macrophages with the same TLR ligands, Pam3CSK4 and LPS desensitizes the TLR2 and TLR4, respectively, and generates TLR tolerance. Moreover, the repetitive TLR stimulation further reduced the production of TNF-α, CCL5, and IL-10 without affecting the constantly augmented level of IL-6 and IL-8 in alveolar macrophages. This may provide alternative explanations for diminished immune responses against the recurrent/repetitive infection by NTHi.

### Altered and abnormal TLR/PRR expression: inaccurate responses to NTHi

The intrinsically reduced expression of TLRs in COPD patients may also contribute to the impaired pulmonary immune response thus facilitating NTHi persistent colonization. Expression of TLR2 or TLR4 are found to be lower on sputum neutrophils, alveolar macrophages, nasal epithelium, and T cells in COPD patients despite high concentrations of IL-8 and MMP-9 (295–298).

The lack of the more protective Th1/Tc1 immune response in COPD patients against NTHi infection might be attributed to upregulated antagonists (A20, IRAK-M, and MyD88s) of the MyD88/IRAK/MAPK signaling pathway in COPD T cells (295). It should be noted that the MyD88/IRAK/MAPK pathway is required for expression of TLR4 in Th1, whereas production of IFN-γ in Th1/Tc1 is TLR4-dependent via the TLR4/TRIF/IKKe/TBK1 signaling pathway. The antagonists prevent the NTHi LOS-induced TLR4 expression in Th1 and Tc1 and thus a reduced secretion of IFN-γ. In addition, unusual high numbers of Tregs in COPD patients have also contributed to effector T cell dysfunction or a Th2/Tc2 predominant immune response (272). However, Freeman et al. reported that Tc (CD8^+^) cells from COPD patients have increased expression of TLR1, TLR2, TLR4, TLR6, and TLR2/1 as well as Tc1 cytokines (IFN-γ and TNF-α) compared to healthy individuals that may imply the auto-aggressive response of lung Tc cells in COPD lung inflammation (299). However, the COPD Tc cells can only be stimulated by ligands for TLR2/1 (Pam3CSK4) yet tolerant to other agonists, indicating the dysfunctional TLRs or TLR tolerance on T cells despite their high level of receptor expression.

Inversely, peripheral blood neutrophils isolated from COPD patients have increased expression of TLR2, TLR4, and NLRP3 (298, 300). Nevertheless, the increased TLRs expression might not improve the microbicidal ability of COPD peripheral neutrophils probably due to the inaccurate responses to cytokines (179). In addition, certain types of SNPs (SNPs) in TLR2 and TLR4 have also been associated with decreased lung function, enhanced inflammatory responses and increased immune cell infiltration in COPD (301). Interestingly, the diminished IL-8 responsiveness of COPD alveolar macrophage to NTHi infection has a strong association with the carriage of TLR9 (T1237C) polymorphism instead of TLR2 (Arg753Gln), TLR4 (Thr399Ile; Asp299Gly), and TLR9 (T1486C) (302). The carriage of TLR9 (T1237C) is also positively correlated with diminished lung function. Of note, the activation of TLR9-signaling cascade in pro-inflammatory cytokine response requires stimulation from microbial DNA (303).

### The tobacco smoke: negative effects on the immune defense against NTHi

The microbicidal malfunction in both innate and adaptive immune cells is also potentially linked to the deleterious effect of tobacco smoke, the major risk factor for COPD. It has been reported that, exposure of tobacco or cigarette smoke can impair phagocytosis/engulfment of NTHi by alveolar macrophages isolated from COPD patients (256, 304). Moreover, the chemical exposure also suppressed the TLR-induced TNF-α, IL-6, and IL-10 production in COPD alveolar macrophages that have been pre-stimulated with TLR2, 4, or 5 ligands (Pam3CSK4, LPS, or phase I flagellin, respectively), or whole NTHi bacteria (305). This may potentially delay the macrophage-dependent bacterial clearance. The suppressive effect of cigarette smoke in macrophage-dependent phagocytosis is due to the suppression of the PI3K signaling cascade which is required for optimal phagocytic activity and movement (256). Meanwhile, the cigarette smoke also inhibits the activation of the p38-ERK signaling pathway and p65/NF-κB, thus dampens the NTHi LOS-induced cytokine production of COPD alveolar macrophages (305). The diminished alveolar macrophage responsiveness could also be related to anticholinergic agents used by COPD patients that results in lower concentrations of NTHi-induced TNF-α (306). Nevertheless, the impaired phagocytosis of NTHi by COPD alveolar macrophages could be improved in the presence of nuclear erythroid related factor 2 and microRNA MiR-328 (307, 308). Interestingly, in addition to the constant exacerbated inflammatory effect observed in different murine model studies, Gaschler et al. observed a rapid pulmonary clearance of NTHi in mice upon exposure to cigarette smoke, and this was positively correlated with an increased neutrophilia in the animal BAL fluid (236, 309–311). However, in other COPD animal studies, cigarette smoke also impaired the IL-22 production that has a potential anti-bacterial activity while delaying the airway clearance of NTHi (311–313). Interestingly, IL-22 might play a protective role in COPD exacerbation as supplementation of IL-22 manages to restore the homeostasis of airway immune response and improve NTHi clearance (313).

The increased airway neutrophilia might be due to the enhanced production of pulmonary IL-17 triggered by cigarette smoke (152, 236, 311, 314). This may imply the important microbicidal role of neutrophils (neutrophilia) in compensating the COPD- or cigarette smoke-associated dysfunctional alveolar macrophages (96, 315). However, such compensation may not be adequate to provide optimal immune defense to eradicate persistent NTHi lower airway colonization, since the cigarette smoke also has profound suppressive effect on the host adaptive immunity, thus constantly risking the COPD patients to episodes of exacerbation and relapsed infection. In adaptive immunity, cigarette smoke impairs the antigen-specific B and T cells responses to NTHi infection. It suppresses the secretion of IFN-γ and IL-4 by NTHi-specific T cells. Antibody production by B cells has also been attenuated, with lower levels of specific anti-P6 antibodies and compromised IgG1, IgG2a, and IgA class switching (311, 312).

A recent and some previous cohort studies revealed that the level of airway antimicrobial cathelicidin (hCAP18/LL-37) in COPD patients increase gradually from the stable disease to exacerbation states (176, 316). Moreover, higher levels of cathelicidin are positively associated with NTHi airway colonization, sputum neutrophilia, and higher concentrations of IL-8, particularly in the NTHi-infected COPD patients. Of note, cathelicidin and other AMPs play important roles in the innate immune defense against different pathogens and persist immunomodulatory properties (317–319). Ironically, it is plausible that the increased level of cathelicidin could diminish or alter the balance in lung microbiota, and the immune/inflammatory response. This might contribute to the “vicious circle,” thus considerably increasing the risk for NTHi infection during COPD exacerbations (214, 320). Moreover, the microbicidal property of cathelicidin could be compromised by the inflammatory conditions in the airway, such as low pH, or the effect of cigarettes that causes peptide citrulination and modification (321, 322). Finally, expression of AMPs (human beta defensin 2 and S100A7) by COPD airway epithelium in response to NTHi infection, is also disturbed by cigarette smoke. The insulted airway cells have also a reduced expression of TLR4 and IL-8, and impaired NTHi-induced NF-κB activation (175, 296). Thus, a large body of evidence exists on the deleterious effects of tobacco smoke.

## Antibiotic treatment of NTHi in COPD

Antibiotic treatment of AECOPD has been shown to significantly reduce the risk of treatment failure, especially for in-patients with severe exacerbations and patients requiring intensive care (323). The efficacity of antibiotic treatment for out-patients with exacerbations is less clear (323, 324).

Recommendations on which empirical treatment to use for AECOPD varies between different countries, but common antimicrobial agents that are frequently used as definitive therapy against NTHi include aminopenicillins (with or without a beta-lactamase inhibitor), tetracyclines, trimethoprim-sulfamethoxazole, and fluoroquinolones. In addition, the Clinical and Laboratory Standards Institute (CLSI) has developed clinical breakpoints for the macrolides azithromycin and clarithromycin (325), whereas the European Committee on Antimicrobial Susceptibility Testing (EUCAST) have not set any clinical breakpoints against this class of antibiotics due to lack of clinical data (326). One study shows that NTHi frequently develops resistance to macrolides during prolonged treatment and that treatment failure may occur, making fluoroquinolones more reliable for eradication in COPD-patients (327). As for aminopenicillins, resistance is also common, with up to 10–20% of NTHi isolates expressing beta-lactamases and an additional 10–20% of the isolates having amino acid substitutions in penicillin-binding protein 3 (PBP3), which reduces their susceptibility to these agents (328, 329). The fraction of isolates expressing beta-lactamases has been stable during the last years, whereas an increase has been seen in isolates displaying altered PBP3 (330, 331). This is worrisome, since some of these amino acid substitutions also confer resistance to third generation cephalosporins (332). Moreover, there seems to be a correlation between isolates expressing altered PBP3 and increased invasiveness. Studies have shown that strains that express a mutated PBP3 with certain key amino acid substitution have a significantly higher rate of invasion of bronchial epithelial cells compared to strains with a wild type PBP3 (333). However, when such mutated PBP3 was cloned into a susceptible wild type strain, invasion efficacy did not increase, suggesting that PBP3 is only indirectly linked to invasion (334).

Besides using antibiotics for acute management of COPD exacerbations, some studies have considered the use of continuous prophylactic antibiotics in the management of patients with COPD (212). There is some evidence that continuous administration of macrolide antibiotics would prevent future exacerbations in a selected population of the most severely ill patients, but a Cochrane review revealed no support for a reduced all-cause mortality or less hospital readmissions (335). However, more recent studies have shown a significant decrease in both the frequency of exacerbations and hospitalizations when long-term azithromycin treatment was chosen (336).

The fact that macrolide antibiotics display not only antimicrobial effects, but also have anti-inflammatory and immunomodulatory properties, has made them interesting to use as prophylactic therapy (212). It has been shown that azithromycin inhibits mucus hypersecretion in the respiratory tract by significantly inhibiting TNF-α induction of the MUC5AC mucin secretion from human nasal epithelial cells (337). More specifically, it has been shown that azithromycin can reduce the NTHi-dependent induction of MUC5AC expression by suppressing the transcription factor activator protein-1 (338). Apart from affecting mucus secretion, it also seems that low-dose azithromycin has the ability to improve phagocytosis of bacteria by airway macrophages (339). One study showed that azithromycin concentrations that were unable to kill NTHi still increased the uptake rate of the bacteria into alveolar macrophages by enhancing their phagocytic function (340). However, the risk of development of antimicrobial resistance limits the use of low-dose azithromycin solely for its immunological properties. This has triggered an interest in finding new macrolide substances that lack antibiotic effect and solely interact with the airway immune system (341).

## Perspective in NTHi vaccine development

The considerable clinical problems caused by NTHi with regard to COPD exacerbations and otitis media has prompted the scientific community to investigate whether a vaccine can be developed against the pathogen (5, 58, 342). The search has been intensified due to a steady increase in antibiotic resistance and a trend of more invasive infections caused by NTHi over the last decade (5). Whereas, a highly efficient glycoconjugate vaccine has previously been developed against Hib, an identical strategy cannot be employed against NTHi due to the lack of a polysaccharide capsule. Vaccine developments efforts have thus been concentrated on identifying NTHi surface structures that are immunogenic, have low antigenic variability, and are conserved across this genetically highly heterogeneous species. Several promising vaccine candidates have been identified in the last 25 years, as excellently reviewed elsewhere (58, 342).

Two of these antigens, fused into one protein, Protein E-PilA, are together with Protein D currently being tested by GlaxoSmithKline in a phase IIb proof-of-concept clinical trial (randomized, observer-blind, placebo-controlled, and multicentric) for infection prophylaxis in COPD patients (50–70 years old) (5). Notably, the *M. catarrhalis* ubiquitous surface protein A2 (UspA2) is also included in the vaccine so that an immune response against both exacerbation-causing pathogens could be elicited by the same preparation. This clinical study (NCT03281876) is the only one currently being conducted on NTHi (and *M. catarrhalis*) according to clinicaltrials.gov, and as the investigations are on-going, the results are currently unknown.

Due to an increase in the difficulty to treat NTHi infections, an efficient and protective NTHi vaccine likely considerably raises the quality of life of COPD patients. Since NTHi-mediated exacerbations contribute to the progression of the disease and a steady deterioration of the pulmonary capacity of those patients, prevention against NTHi infections potentially slows down the debilitating effect of the disease. It is therefore critical to continue this line of research until such a vaccine has been obtained. It could also be worth targeting non-conventional structures with a vaccine, such as the secreted enzymes urease and IgA1-protease that have proven important for NTHi infections in COPD patients in several studies (222).

## Conclusions

COPD is a multifaceted airway disease. Several factors influence the clinical outcome of COPD. Importantly, the crosstalk between intrinsic factors (the stability and integrity of the airway immune response and structure in addition to hereditary factors), and the extrinsic factors (lung microbiome, viral and bacterial infections, meteorological factors, and noxious inhalation) determines the fate of lower airway opportunistic infection by *H. influenzae*. Intriguingly, NTHi has been one of the most isolated pathogens at both stable and exacerbation states of COPD. Such persistent airway colonization of NTHi costs virulence fitness to counteract with the bactericidal effect of the host immune response. Adversely, the impaired defense mechanisms in COPD are not only unable to protect the lung structure from inhaled physical assaults, but they also fail to suppress NTHi infection. The disoriented immune response in COPD instead allows the pathogen to cause more harm and inflammation in the airways. The currently used bronchodilator and inhaled corticosteroid therapies have limited efficacy in preventing disease progression in COPD. Moreover, the inhaled corticosteroid therapies might have side effects that may weaken the immune response. Hence, more investigations are needed to garner a more adequate knowledge regarding the variabilities in immune networking of COPD. This knowledge will be an important platform for a more efficient drug design. In addition, a vaccine targeting NTHi is another important approach in controlling the infective exacerbations in COPD as the antibiotic treatment is also getting dampened by the emergence of NTHi antibiotic resistance.

## Author contributions

Y-CS coordinated and drafted the major part of the manuscript, and prepared the figures; FJ participated in the literature study of virulence and vaccine research of NTHi; JT prepared the review section for NTHi epidemiology in COPD and antibiotic studies; Y-CS and KR edited and critically revised the manuscript. All authors read and approved the final manuscript.

### Conflict of interest statement

The authors declare that the research was conducted in the absence of any commercial or financial relationships that could be construed as a potential conflict of interest.
